# At the Origin of Animals: The Revolutionary Cambrian Fossil Record

**DOI:** 10.2174/13892029113149990011

**Published:** 2013-09

**Authors:** Graham E Budd

**Affiliations:** Dept of Earth Sciences, Palaeobiology, Uppsala University, Villavägen 16, Uppsala, Sweden, SE 752 36

**Keywords:** Cambrian explosion, Ediacara, Animal evolution, Developmental evolution.

## Abstract

The certain fossil record of animals begins around 540 million years ago, close to the base of the Cambrian Period. A series of extraordinary discoveries starting over 100 years ago with Walcott’s discovery of the Burgess Shale has accelerated in the last thirty years or so with the description of exceptionally-preserved Cambrian fossils from around the world. Such deposits of “Burgess Shale Type” have been recently complemented by other types of exceptional preservation. Together with a remarkable growth in knowledge about the environments that these early animals lived in, these discoveries have long exerted a fascination and strong influence on views on the origins of animals, and indeed, the nature of evolution itself. Attention is now shifting to the period of time just before animals become common, at the base of the Cambrian and in the preceding Ediacaran Period. Remarkable though the Burgess Shale deposits have been, a substantial gap still exists in our knowledge of the earliest animals. Nevertheless, the fossils from this most remarkable period of evolutionary history continue to exert a strong influence on many aspects of animal evolution, not least recent theories about developmental evolution.

## THE CAMBRIAN NOW AND THEN

The Cambrian Period, first proposed in 1835, has had a long and controversial history. Adam Sedgwick the Cambridge geologist who first proposed it, worked on deformed and largely unfossiliferous rocks in the north of Wales, but within a few years it became clear that the fossiliferous parts of his system overlapped with the next period up, then the Silurian [1]. With no fossils to support it, the rest of the Cambrian was nearly stillborn, and indeed in the first edition of the *Origin*, Darwin only refers to the Silurian. Nevertheless, true upper Cambrian fossils were found in Britain in the 1840s, mirroring discoveries elsewhere including in Scandinavia and Bohemia. In 1862, whilst on holiday in Pembrokeshire, Wales, the noted British geologist John Salter steered his boat into the small inlet called Porth-y-Rhaw, mistakenly thinking it to be the harbour of the nearby village of Solva. Here he discovered the large trilobite *Paradoxides davidis* [2], thus making certain the presence in Britain of a fauna known for some years, also from Bohemia (from the work of Barrande) and Scandinavia. This so-called “Primordial” fauna was at the time thought to be the oldest in the world, and its discovery in Britain was considered to be so important that a *Paradoxides* discovered subsequently became “No. 1” in the new arthropod catalogue of the Natural History Museum of London. In the next two decades, it became clear that an even older fauna than these (presently) middle Cambrian fossils was to be found around the world. Altogether, these three faunas made up the traditional “lower”, “middle” and “upper” Cambrian formalized by Charles Walcott in 1890 [3]. Confidence was expressed that the base of the fossil record of life had been achieved on various occasions, and it seemed to be marked by the appearance of those most iconic of fossils the trilobites, in rocks that we now consider to be approximately 520 million years old. 

At each stage of this process, the oldest known fossil record was implicated in discussions about the origin of animals. Ironically, however, all early instances were in fact dealing not with the oldest records of animals, but with fossils much younger than what are now known to exist. Darwin, for example, has a famous passage in the *Origin* about the sudden appearance of certain groups of organisms – including the trilobites – in the fossil record; but essentially many of the fossils he is talking about there are what would now be considered to be *Ordovician* in age. A further surprise came after the Second World War, when the results of much work in Siberia by Soviet stratigraphers became available in the west (e.g. [4]), showing that richly fossiliferous rocks lay beneath those bearing the oldest trilobites – thus confirming the insights of Matthew [5] who worked in New Brunswick at the close of the 19^th^ century, but whose work fell into disregard.

The advent of reliable radiometric dating methods in the last two decades has allowed a timescale to be placed on these early events, and this has held various surprises (e.g. [6, 7]). In particular, it seems that this very early Cambrian time before the trilobites appear in the record is much longer than previously suspected. This in turn means that a degree of temporal resolution in the events of the Cambrian is now possible – although what these events mean remains debatable.

**Fig. (1):**
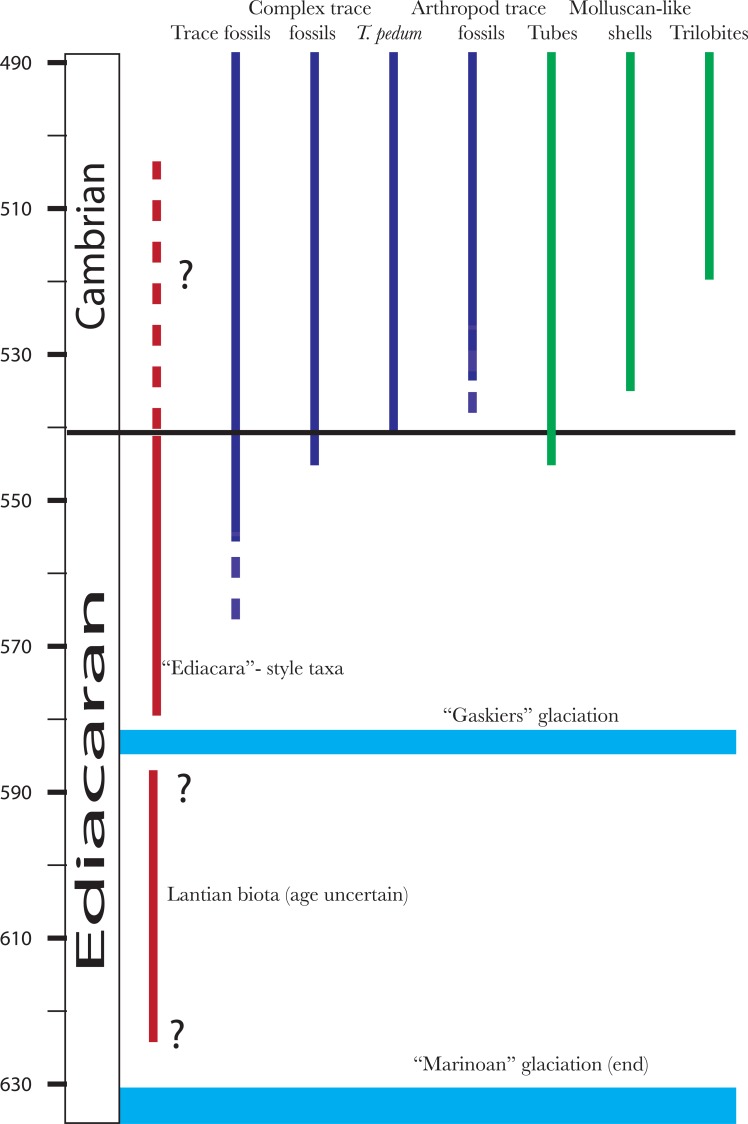
A broad outline of important aspects of the fossil record from the Ediacaran to Cambrian. Dating from e.g. Bowring *et al.* 2007. *T. pedum* is the trace fossil that formally marks the base of the Cambrian.

The popular view of the so-called Cambrian explosion is still heavily skewed by the most important windows into ecology and diversity during this time – ie the famous exceptionally-preserved faunas, particularly the Burgess Shale ([8, 9]), but also, increasingly, the Chengjiang Biota [10]. To these deposits can also be added perhaps secondary but still highly significant biotas such as the Greenland Sirius Passet and the Swedish (and elsewhere) “Orsten” fauna. These and other biotas reveal a rich array of Cambrian life – animals and algae from which it can be seen that a recognizable marine ecology was running by the time these rocks were deposited [11-13]. Of particular interest is the rich evidence for predation known from the record [14]. All these deposits are however considerably younger than rocks now considered to belong to the base of the Cambrian. Precise dating of rocks still remains somewhat uncertain, but it seems unlikely that any of these exceptionally-preserved biotas is much older than about 516 Ma – ie some 25 million years younger than the base of the Cambrian at perhaps 541 Ma (Fig. **1**).

The base of the Cambrian itself is to some extent arbitrarily chosen, but in principle marks significant turnover in animal ecology. The general guide for many years, when its position was under discussion, was to place “Ediacara”-style fossils (see below) in the Precambrian, and animal-like trace fossils in the Cambrian. However, the ranges of these two suites can now be clearly seen to overlap. Older ideas of a sort of barren “no-man’s land” lying between the diverse Ediacaran and Cambrian biotas have collapsed under the pressure of new dating [6], and this in turn suggests that the two biotas should be considered jointly. In other words, when discussing the Cambrian explosion, it seems almost inevitable to turn attention backwards to the Ediacaran as well as forward to the Recent, problematic though it is.

## NEW DATA FROM AROUND THE WORLD

In the last thirty years or so, a remarkable amount of new data has become available with direct bearing on the nature of the Cambrian explosion. It can be divided into several broad categories: the conventional fossil record of the Cambrian; exceptionally preserved fossils; discoveries in the Ediacaran, and the input of molecular and isotopic results.

### An Extended Cambrian Record

As discussed above, the fossil record of animals was for many years considered to commence very close to the age of the first trilobites, and indeed, this point in the record was a constant contender for marking the base of the Cambrian. However, it is now clear that trilobites appear relatively late in the record. Pre-trilobite animal fossils were probably first known from Massachussetts [15] but the most significant discoveries were made after WWII in Siberia, especially along the banks of the Aldan and Lena rivers in south Siberia [4]. The Siberian platform hosts a vast extent of mostly carbonate rocks covering the Ediacaran-Cambrian interval that are widely distributed across its approximately 3.5 million km^2^ extent. In the south, the best investigated area, it became clear that a considerable thickness of pre-trilobite rocks contained a rich diversity of generally small fossils preserved in phosphate, which became known as the small skeletal fauna, or “small shelly fossils”. The most diverse faunas were found in a series of rocks termed the “Tommotian”, and because of their richness these were considered to be Cambrian in age. Beneath them lie much more poorly fossiliferous rocks, however, which have been variously termed the “Nemakit-Daldynian” or “Manaykaian”. The dating of these rocks, and how they correlate to other sequences around the world, have proved to be highly controversial (e.g. [16]), but it is clear, especially from sequences further north in Siberia where radiometric dating has been possible, that almost all of these rocks are of Cambrian age when compared to the international base of the Cambrian on Newfoundland. Our view of the sequence of events in the Cambrian has thus been transformed in the last few decades, with the basal rocks now being known to contain a suite of trace fossils of moderate diversity, and an increasing diversity of small shelly fossils, including a large number of tubes, plates and cap-shaped fossils (e.g. [17]). The affinities of these early fossils are much debated as soft parts are unknown, but at least molluscs seem to represented.

### Exceptional Preservation

As well as the considerable extension of the conventional fossil record, a series of very important exceptionally preserved biotas have been discovered. The Burgess Shale, and rather similar biotas in North America found in the early years of the twentieth century, was complemented by successive finds in China (the Chengjiang fauna) and North Greenland (the Sirius Passet fauna). The Chengjiang biota in particular is remarkably diverse and yields many examples of taxa almost unknown from the rest of the fossil record such as putative tunicates [18] and a remarkable array of apparent early deuterostomes [19].

### Small Carbonaceous Fossils

For decades, researchers have been extracting small, sometimes fragmentary fossils preserved as organic carbon, from siliceous rocks using hydrofluoric acid (HF). The characteristic fossils recovered in this way – termed acritarchs – are a heterogeneous assemblage of pro- and eukyarotic taxa that probably include cyanobacteria, green algae, and other related groups, and their record extends deep into the Precambrian (e.g. [20]). More recently, remarkably-preserved organic fossils have been recovered using somewhat gentler HF methods from Cambrian rocks (e.g. [21]), and these promise to extend and complement the Burgess Shale-type material. The most significant find is of fragments that can be confidently assigned to crown-group crustaceans [22], and this has extended the record of many of these groups back at least to the middle Cambrian. All forms of preservation have pluses and minuses, and the exquisite nature of these fossils must be balanced against their invariable lack of completeness, but it is clear that this area of research offers a completely new insight into the taxa that were generated during the Cambrian explosion and their ecological significance.

## ISOTOPES AND DATING

One of the hindrances to a full appreciation of the Cambrian explosion has been the difficulty involved in accurate dating. In general, rocks are dated in two broad ways: with relative methods including biostratigraphy, that aim to establish the *order* of events in a particular place and then correlate this with other rocks around the world, and *absolute* methods using the decay of radioactive elements (notably the decay of uranium to lead) to put these correlated series into a real time frame. For a variety of reasons, both these approaches have traditionally been extremely difficult in Cambrian rocks, especiallly from the lower Cambrian. 

The biostratigraphy – the relative appearance of biotas – continues to be problematic, partly because of inconsistent and sometimes rather low-quality taxonomy of lower Cambrian taxa (thus making the true ranges of the organisms that really existed hard to determine), and partly because of the peculiar nature of lower Cambrian biotas in general. Many of them consist of endemic taxa that are of no use in making age comparisons across regions or continents. As a result, the formal stratigraphy of especially the lower Cambrian is still rather fluid, despite significant recent advances in the area. Conversely, absolute radiometric dating has also been difficult because the most reliable methods require volcanic rocks with particular minerals, in particular zircon, for dating, and these are in short supply in this stratigraphic interval. It should also be noted that even where these types of rock are available, must be relied on indirect methods for their dates to be propagated around the world. These problems have been somewhat alleviated in recent years by an increasing recognition of another type of correlation based on variation in the stable isotope composition of especially carbon (e.g. [17]). The period of time from just before to just after the base of the Cambrian was one that witnessed large variations in the relative amounts of ^13^C and ^12^C, and these variations have been assembled into a standard curve that can be used for correlation purposes. A particularly exciting recent development has been to calibrate the absolute age of this carbon isotope curve, using the co-occurrence of rocks suitable for isotopic evaluation with volcanic ash-beds lying within them, most particularly in Morocco. This dated carbon curve has been used to set up a global reference for faunas in many area of the world, especially in China, Mongolia and Siberia. In detail, this method is not free from difficulties, and some of the results have been somewhat surprising (for example, the rocks in Mongolia, which bear a rather standard set of fossils, have been suggested to be somewhat older than similar rocks in Siberia), but the broad outlines of the sequence of events, and their timing, are now becoming somewhat clearer [17].

## MOLECULAR DATING AND FOSSILS

Another area of both advance and controversy is that concerning molecular dating. This includes two broad areas: calibrating the accumulated differences found in molecules of living organisms to determine their time of origin, and using molecules found in the fossil record as proxies for the existence of the organisms known to produce them. Although the first attempts at “molecular clock” methods are over 40 years old [23], the recent subject really dates from a flurry of papers in the 1990s, most of which suggested that the animals started diverging well before the base of the Cambrian, perhaps some 1200 Ma. In recent years, molecular clocks have come under a great deal of scrutiny, and the present consensus is that the bilaterian animals can be dated by molecular clock methods after all to have arisen shortly before their fossil record commences [24]. Recent results still suggest, however, that at least the sponges arose some 200 Myr before the beginning of the animal fossil record. This builds on the other aspect of molecular dating of animal origins, that of finding direct molecular fossils of taxon-specific molecules. In particular, ref. [25] reported demosponge-specific sterols from before about 635 Ma, and suggested this implied a deep cryptic history of the sponges. Demosponges, together with their sister group the hexactinellids, possess siliceous spicules, and if as seems reasonable their spicules are homologous, then the origin of spicules must predate that of the demosponges – ie some time before 635 Ma, a result also suggested by a molecular clock method. However, these results, based as they are on tying a molecule that might have had a broader distribution in the past to a particular living clade, have not persuaded everyone of the stronger result of sponges really having emerged that long ago [26]. Living sponges in general possess small mineralised spicules that have several ways of being preserved [27] and the apparent total absence of spicules from the fossil record until at the very least very close to the base of the Cambrian [28] renders this early origin suspect. 

**Fig. (2):**
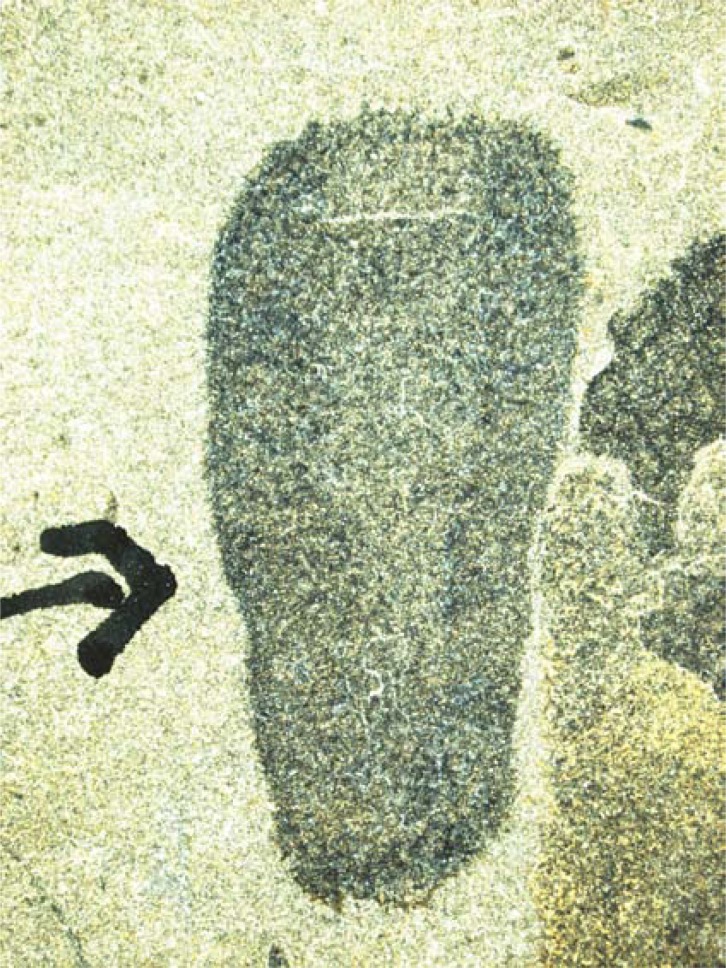
A typical fossil sponge from the Middle Cambrian Burgess Shale, *Hazelia delicatula*. The fossil is approximately 9mm in length. Photograph courtesy of Joe Botting.

Another highly problematic aspect to understanding the earliest history of the animals is that the relationships of the extant forms are highly unclear. Traditionally, it was acknowledged that the sponges, cnidarians and ctenophores were basal in the animal tree (later to be joined by a couple of other groups the placozoans and myxozoans), and that the sponges were the most basal, with cnidarians and ctenophores forming a “coelenterata” group as sister-group to the bilaterians. However, this view was attacked by morphologists who argued that the cnidarians and ctenophores did not belong together; and, more recently by a series of rather remarkable molecular results that place ctenophores at the base of the tree, and make the sponges paraphyletic [29], a result that in combination with molecular results placed sponges deep in time. These results are to a greater or lesser degree problematic. The greatest surprise is the consistently basal position of the ctenophores which have apparently bilaterian features such as mesoderm with muscle and nervous tissue. If the sponges are paraphyletic, they represent a grade that gave rise to the cnidarians and bilaterians, which in one sense would be very helpful as we would know almost exactly what genomes the earliest “higher” animals had. However, more recently it has become evident that the position of none of these groups is resolved, with one recent analysis recovering the traditional coelentorata and basal monophyletic sponges ([30, 31] – see also [32] which comes to similar conclusions ). Sponge paraphyly creates some problems for the fossil record as it makes the deep origin of the fossilisable spicules even more likely. Although sponges have been claimed from very deep in the fossil record, including from the oldest known Ediacaran assemblage (see below), no sponges are universally accepted until the base of the Cambrian is reached when spicules start appearing in the record (Fig. **2**). Nevertheless, given that the sister group to the animals is the Choanoflagellata, which closely resemble the so-called “collar cells” of extant sponges [33], which in itself raises interesting questions about the origin of the animals as a whole, it seems likely that something *like* a sponge grade of organisation lies at the base of the animals, whether or not the living sponges themselves are para- or monophyletic.

## NEW PERSPECTIVES ON EDIACARAN FOSSILS

As the base of the Cambrian became reliably dated (e.g. [6, 7]), its age relationship to the underlying Ediacaran rocks and their perplexing biotas became clearer. Dates for the Ediacara- style biotas are surprisingly poorly documented, but a date of about 565 Ma in Newfoundland [34] is commonly cited for the major biotas at Mistaken Point. For some years, this assemblage was considered to be the oldest known of Ediacara organisms, but discoveries of well-preserved fossils some 1.5 km stratigraphically below this point in the same region [35] suggested that the their first appearance was considerably older than this (a date of 579 Ma is quoted by [36]), perhaps close to the date of an important glacial interval, the Gaskiers, at around 582 Ma [37]. This older assemblage has yielded some remarkable taxa, and has extended the range of several important Ediacara taxa by 15-20 Myr. These include both the huge frond *Charnia wardi *[35], some specimens of which are over 2m in length, as well as an assemblage of what appear to be juvenile specimens of *Charnia masoni* [38]. *Charnia masoni*-like fronds are also known from the White Sea area from about 549 Ma [39, 40], making this an extremely long-lived morph. Rather than representing a relatively short-lived burst of “failed evolutionary experiments”, the Ediacara-like biota was both long lived and cosmopolitan. Another taxon of interest from this very early assemblage is *Thectardis* which, although lacking specific morphological details, has been suggested to be a sponge [41]. Given the paucity of details this fossil possesses, and despite the biomechanical arguments in favour of its poriferan affinities [41], a certain degree of caution is surely in order before this claim is accepted uncritically. More recently still, a biota from the Lantian Formation of South China has been suggested to be almost certainly older than this assemblage from Newfoundland [42]. It contains some macroscopic taxa that are probably algal in affinity, but also a more diverse range of organisms that are more intriguing. Indeed, one form (Fig. **3E** of [42]) has recently been suggested to be related to the conulariids, a group of Cambrian problematica that are often compared to cnidarians [43]. If the dating and affinities can be sustained, this would suggest that both stem-group cnidarians and bilaterians were present almost all the way through the Ediacaran Period (635-541), which would be a signficant advance in our our understanding of early animal evolution. Once again, this is an area that would be worthy of further investigation.

The disappearance of the Ediacara biota is problematic [36]. Although the fossil record superficially gives the impression that Ediacara-type taxa undergo an abrupt “mass extinction” just before the base of the Cambrian, alternative models, that they were progressively competitively displaced by the emerging metazoan clades, or even that their disappearance is largely owing to preservational changes [44] are both possible. Did the Ediacarans disappear as the result of some sort of catastrophe, and was the resulting vacant space taken over by the rapidly-expanding metazoan clades, or did they progressively disappear as metazoans, above all by burrowing and thus profoundly altering the nature of the seafloor and carbon cycle, remove the environments within which the Ediacaran clades flourished, the sort of ecological interaction known as trophic amensualism? At present, the data available cannot fully constrain these models, although clearly the advent of mobility had a marked effect on substrates [36]. Nevertheless, the nature of the succeeding Cambrian period offer some intriguing insights into what ecological processes were driving the diversification during this time. In particular, the argument has been made that a mobility developed within the bilaterians and they started to burrow, the very environment in which the ediacarans lived was destroyed, a change called the “Cambrian substrate revolution” [45], the exact importance and timing of which, however, remains problematic.

What, then, does the Ediacaran-Cambrian interval represent in terms of animal evolution? A conventional view might be that directly after the global glaciations represented by the Gaskiers deposits in Newfoundland and elsewhere, rising oxygen levels enabled the first appearance of large, multicelllular organisms (ie the oldest Ediacara-style biota) at around 580 Ma. By about 550 Ma or so, some of the Ediacara-style biota (e.g. *Kimberella* [46]) had developed movement and had a variety of different ecologies. At this point, two more or less distinct views can be distinguished. The first is that these “higher” Ediacara taxa were already representatives of familiar animal groups such as annelids, placozoans, molluscs and arthropods, and as a result, the ensuing Cambrian explosion did not in fact have to generate many major new clades – rather, it represents a diversification of taxa already present (the view most notably expounded by [47]). For example, if one considers that a taxon such as, for example, *Spriggina* represents something like a stem-group annelid, then its presence implies that both the deuterostomes and ecdysozoans have already diversified, along with many other lophotrochozoans. The second common view is that the Ediacarans represent a sort of evolutionary “dead end” that was eliminated by the rising tide of bilaterians, again enabled by increasing oxygen levels. 

I remain doubtful that any known Ediacara-style organism represents a crown-group bilaterian, despite the increasing trend in the literature to consider at least some to be so. The strongest counter-argument is a phylogenetic one combined with the trace fossil record. If a relatively advanced bilaterian such as a stem- or crown-group annelid, mollusc or arthropod is present in an Ediacaran assemblage, then one can reasonably ask where all the other crown or stem-group annelids, molluscs, arthropods, deuterostomes etc that logically must be present are – in other words, the presence of a single diagnosable bilaterian would imply that a substantial radiation of the Bilateria had already occurred. If the answer to this is that many of the Ediacara-style taxa looked very different from members of modern clades, then one might ask why the living phyla share so many features, such as a through gut, muscles, nephridia, blood vascular systems, and so on. If there are so many putative bilaterians around, why is it that only a tiny handful of Ediacara-style taxa show any even faintly plausible animal features? In other words, this view of evolution repeats the old conceit that the evolution of each of the crown-group phyla – distinguished only by the fact that some members happen to be alive today – can essentially be treated in isolation from that of all the others and does not need to make reference to their putative ancestral features, a point I shall return to below when considering the genetics of the radiation of the Bilateria. In fact, apart from their shared features such as muscles and the through gut at minimum, the stem groups to several major clades are now slowly being elucidated (e.g. [48, 49]). Put simply, taxa such as *Spriggina* do not look like known reconstructions of stem groups to taxa such as molluscs, annelids and arthropods. 

Another problem is provided by the trace fossil record. The simplest horizontal trace fossils are known from about 555 Ma or perhaps a little earlier [50], and by about 545 Ma in Namibia, somewhat more complex forms start to appear ([51]; cf. [52] for a report on the diversity of Ediacaran trace fossils). Recent claims of earlier traces [53, 54] stand presently in need of further investigation [55]. As the Ediacaran-Cambrian boundary is crossed, these trace fossils diversify greatly, and within a few million years, lineage-specific traces such as *Rusophycus* (arthropods) appear [56].

What does this diversification of trace fossils represent? If any Ediacara-style fossils represent bilaterians, then it follows that this diversification cannot represent the diversification of bilaterians *per se*, only a sub-set of them. Yet, which ever subset or subsets it represents, the bilaterians must *already* have diversified up to that point without previously leaving a trace fossil record, even though it is likely that many of these basal forms were complex and large [57], had through-guts, musculature, and so on – in other words, were ideally suited for burrowing. Given that at least *some* animals were burrowing at this time, why not more? In this scenario, rather than representing a true diversification of behaviour through time, the trace fossil record would reflect the multiple and independent exploitation of sediment by many different lineages through time. But if this was the case, it is difficult to know why it starts simply and rapidly becomes more complex. Although a somewhat analogous case can be made for diversification of trace fossils after major extinction events such as the end-Permian crisis [58], where tiering starts shallow and progressively deepens after the crisis, the pattern of diversification itself across the Ediacaran-Cambrian is not really similar in terms of the morphology of the burrows themselves. In other words, when we have control over whether or not bilaterians existed before an invasion of infaunal niches, the pattern of when they do pre-exist does not really match what is seen in the Cambrian. 

Nevertheless, the fact that one radiation of large organisms (those seen in the Ediacara-style asemblages) overlaps with another (the Cambrian explosion) after about 1 Ga of having eukaryotes without any such ecologies strongly suggests a linkage between the two. The conventional view is that this proximity in time was enforced by general amelioration of the environment, ie by an increase in oxygen levels [59]. Whilst oxygen levels do seem to have increased towards the end of the Ediacaran [60], there are cogent reasons for doubting it being the primary control in bilaterian radiations [28, 61, cf. 62], not least because there is a problem in understanding the cause-and-effect direction. [61]. Another suggestion has been that benthic ecosystems diversified because of more efficient and concentrated export production of organic carbon as the result of the introduction of the zoological mesoplankton [63]. Whilst the establishment of such a system was doubtless important, and the fossil record has been invoked for direct evidence for its existence in the Cambrian at latest, at least two significant problems need to be overcome before such a view can be considered as established. The first is that the remarkable small organic fossils that were taken to imply the presence of a mesozooplankton seem to belong to more “ordinary” sized organisms [22], and therefore cannot be taken as direct evidence for the presence of such a system. More seriously, this scenario, like many that hope to explain the Cambrian explosion, seems to rely on the presence of a particular set of taxa (ie the crustaceans and other mesozooplankton) that were themselves the *product* of the Cambrian explosion (and a relatively derived one as well) and thus cannot be the *cause* of it. 

With this ecological perspective, can anything more be said of the vexed question of Ediacara assemblage relationships? Increasingly firm control on the dating of Ediacaran assemblages (reviewed in [36]) suggests a definite temporal distribution of the various clades they identify. The oldest assemblages in Newfoundland are dominated by “rangeomorphs”, although, rather curiously, *Charniodiscus*-like fronds are also present. In the later, “White Sea” and “Nama” assemblages, apparently bilaterally symmetrical and (presumably!) non-frondlike taxa such as *Spriggina* appear, and these are joined by the truly complex *Kimberella *[64]. An obvious explanation of this distribution, is that this diversification truly represents a radiation of basal animal groups, from sponge-grade at the bottom (rangeomorphs and potential sponges themselves) through stem-group eumetazoans and stem-group bilaterians. What makes this picture so difficult to elucidate is not just the enigmatic nature of the Ediacara-style taxa themselves, but also the profound problems associated with understanding the relationships of extant taxa. If we had some ideas about what stem-group eumetazoans (representing a transition from a sponge to an animal close to the last common ancestor of cnidarians and bilaterians) or stem-group bilaterians actually looked like, perhaps the Ediacara assemblages would be relatively unproblematic. Two issues stand out as being particularly problematic: the placement of the ctenophores, and the evolutionary role of the placozoans, which have been suggested to shed light on the affinities of *Dickinsonia *([65]. Classical morphological assessment of the ctenophores has variously placed them as deuterostomes [66], as the sister group to cnidarians, or to bilaterians, or to eumetazoans. However, molecular data including genomic-level analyses persistently place them at the base of the animal tree, either as the sister group to all other animals, or as a clade within the sponges [67]. Placement almost anywhere else on the tree would break up one of the problematic morphological long branches: if they truly belong to the base of the tree (a notion that is likely to be resisted by morphologists for some time, however), then almost all of their morphology is likely to be convergent with that of the eumetazoans. Placozoans represent another enigma, because of their tiny size and peculiar amoeba-like qualities. Recent molecular work [30-32] suggests that they are the sister-group to eumetazoans (cnidarians and bilaterians), but their exact significance remains obscure – a recent claim that they have retained an ancient mode of feeding seen in the Ediacaran taxa is interesting [65] but cannot be well supported at present. 

## DEVELOPMENTAL EVOLUTION AND THE CAMBRIAN EXPLOSION

In the late 1980s and early 1990s, as the impact of Steven Gould's book on the Burgess Shale, *Wonderful Life* [8] became felt in the broader biological community, and as the molecular basis for animal development began to be elucidated, the attractiveness of trying to tie the two together became irresistible (e.g [68-70]). Gould's view was that the Cambrian exceptional record revealed an unparalled explosion of different body plans, giving rise to a substantial diversity and disparity that was then pruned by later extinction. The problem with this view is that it has since become apparent that, rather than representing entirely distinct clades, most if not all of the known record represents stem groups to living groups (e.g. [48]). As a result, the Gouldian view has largely faded from the palaeobiological literature, even if it lingers elsewhere. Since then, views have thus grown somewhat more sober, but the interactions between the palaeobiology of the Cambrian explosion and developmental evolution remain of great interest. In particular, the pattern of diversification and what it means for the assembly of the genetics of development is under intense scrutiny. Once again, two broad pictures can be distinguished. The first is powered by the recognition that many of the key genes now involved in animal development were present much deeper in the animal tree than previously expected [71]. In addition, the genes involved in some key animal features do not seem to be particularly well conserved functionally (e.g. [72-74]), leading to suggestions, for example, that striated muscle evolved independently in bilaterians, cnidarians and ctenophores. However, these suggestions rely on the idea that developmental genes, or even constituent proteins, are reliable markers of homology, and this *need* not be the case [75]. Conversely, some of the genes involved in the construction of organ systems like muscles and the nervous system can be seen to have very deep roots (for discussion of possible precursors to nervous systems, see [76, 77]. A story of independent co-option of key molecular machinery lying behind adaptively evolved innovations seems thus to be suggested. This naturally raises the interesting question of how this machinery became assembled in the first place, and what was its undoubtedly different function whilst this was taking place. 

Secondly, it is quite often claimed (e.g. [78, 79]) that the *pattern* of the Cambrian explosion shows a qualitatively different *process* must be behind it when compared to the adaptive radiations of today. In particular, it is argued that the pattern of Cambrian evolution is very “top down” - ie, the phyla diversify first, followed by orders and so on. This gives rise to a pattern in the Cambrian faunas that has been noted for several decades (indeed, it was commented on in the 19^th^ century), that they appear to be very “top heavy” taxonomically – many phyla and classes, and relatively few genera and species in each. As Davidson and Erwin ([80], p. 796) put it, “What mechanisms account for the fact that has there has been so little change in phylum- and superphylum-level body plans since the Early Cambrian...though on the other hand, great changes have subsequently occurred within phyla and classes (e.g., the advent of tetrapod vertebrates, insects, dinosaurs, modern forms of echinoids, and cephalopods)?”

**Fig. (3):**
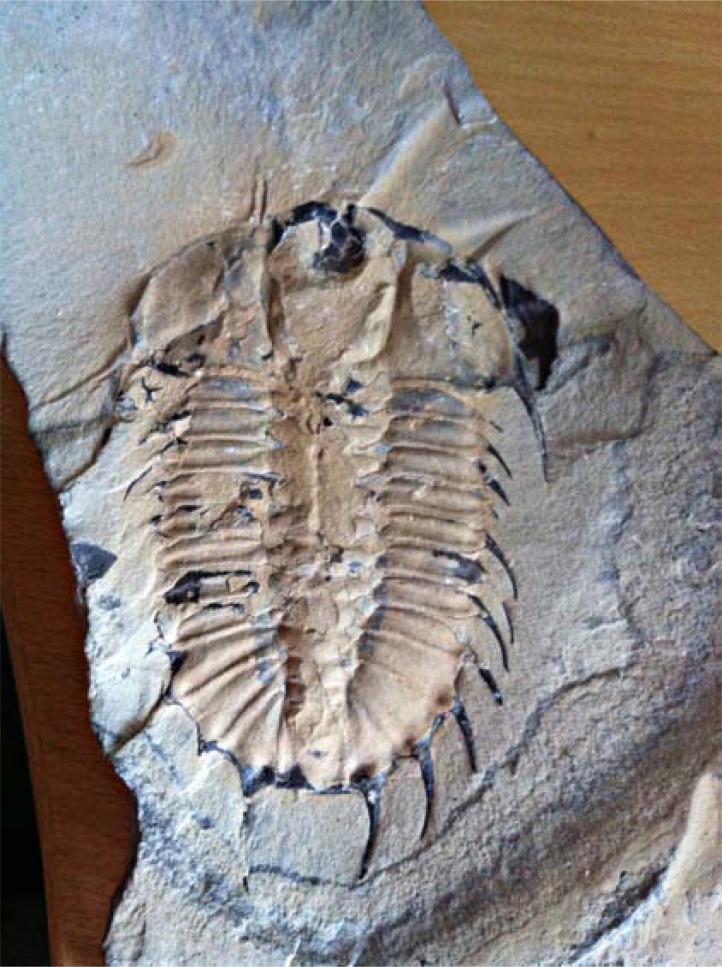
*Kootenia* sp, a typical trilobite from the Middle Cambrian of Greenland, a product of the enormous radiation of trilobites that took place around 520 Ma. The body is approximately 87 mm long.

**Fig. (4):**
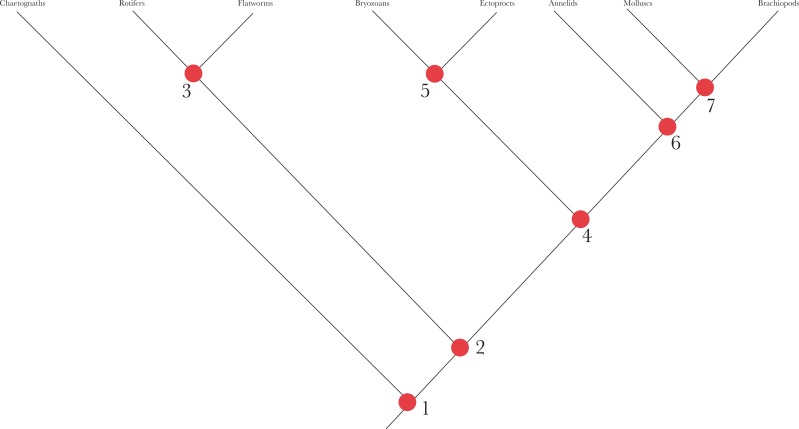
A simplified phylogeny of the Lophotrochozoa (or Spiralia), after [97]. Each of the numbered nodes subtends a number of phyla, and thus the developmental genetic state at each of them cannot have constrained future evolution within the total clade at which base each node lies. If any genetic constraints exist that steer future evolution of the phyla, then they must have evolved within the stem-groups to each phylum, and not be shared in common (e.g. like the kernels of [80]). However, then it is difficult to understand why there would be a persistent asymmetry in pairs of branches such as Branch A and Branch B, wherein only branches that give rise to phyla of today develop constraints.

There are several problematic aspects to this claim. The first is that there are no objective measures of what classes, orders etc are, so making a side-by-side comparison of their numbers through time is difficult. An approach has been made by studying so-called “disparity” amongst different clades (e.g. [81]) that in some clades does seem to show sparse occupancy of morphospace in the Cambrian, a measure that is independent of taxonomic assignment and rank. However, if there *is* such a pattern, it is not clear that it is always adhered to. The trilobites, for example (Fig. **3**), undergo an enormous radiation of rather similar taxa within a short period – almost 600 genera and some thousands of species are known in the lower Cambrian alone, which gives only about 10 million years for this radiation to take place (see data in [82]). Third, it is unclear exactly what claim is being made here. The idea of gene regulatory network (GRN) “kernels” [80] has been sometimes used to imply that the “body plan” features of super-phyla and phyla – mesoderm, blood vascular system etc evolve first, and the “small” features later, and that the reason for the later status of the early features is that the GRN networks for these features become effectively locked in in highly refractory states that persist for the rest of the Phanerozoic. These authors note that there is a broad (but admittedly not perfect) correspondence between the level of communality within the Linnean hierarchy and the level within the developmental hierarchy. What, however, does this mean in practice? If one considers how on a phylogenetic tree such developmental features might plot on, several problems immediately become apparent (it is perhaps telling that no-one seems to have attempted to do this). Insofar as GRNs can be identified as being conserved (ie shared across living phyla), the implication surely is that they were present in the last common ancestor (LCA) of these phyla. But if so, they were not being assembled *per se* within the stem-lineages of those phyla, and thus it is hard to see in what sense the GRNs constrained phyletic evolution. For if the presence of the kernel constrained evolution of the phyla that possess them, then why did it not also constrain the lineage leading to their LCA and that gave rise to the disparity of both? This issue comes about because the living phyla have themselves a hierarchical and branching relationship with each other as well as to their larger groupings, and thus the last common ancestors of different pairs of phyla will have different features, morphological and genetic. Consider, for example, the relationships of the Lophotrochozoa – although not particularly well-resolved, a clear hierarchical structure amongst its contained phyla can be distinguished (Fig. **4**). Each of the contained phyla allegedly has its own “body plan”, but one can see that the regulatory changes associated with them must have a nested distribution. In other words, as one passes up the Lophotrochozoa away from its LCA, its GRN structure should be getting more and more constrained and “kernelised” - but one surely does not not see this supposed increasing constraint as the phyla furthest away from the LCA - e.g. the annelids, molluscs etc are approached (Fig. **4**). This can be seen in another way, by considering the alleged asymmetry of constraint at each node. If the argument is that the inherited genetic structure constraints later innovation, then at a node subtending one phylum as one branch and a group of (diverse) phyla as another, one can see that whatever is causing the alleged constraint, it cannot be the inherited genetic structure at that particular node – because one branch leading from it diversifies greatly (ie into a group of phyla – Fig. **4**). Another problem is that it is the features of the largest groupings such as the super-phyla that, far from being conserved, seem to be highly variable (e.g. [83]). Within “protostomes”, for example, one can see enormous variation within how the coelom is formed, how gastrulation takes place, whether development is indirect or direct, and so on [57, 83]. Or to take another example, the clade consisting of annelids, molluscs, brachiopods and phoronids; despite being embedded within the Lophotrochozoa, this clade shows enormous variation, being held together on morphological grounds largely on embryological details. One can also note that even the earliest animals did not consist of “just” super-phyletic or phyletic characters – they also had all the details of functional organisms such as colour, bristles, peculiarities of feeding and structures and so on [84]. The developmental engines of both high and low-level taxonomic features must thus have developed hand-in-hand. Finally, one can object to the idea of evolutionarily intractable developmental systems on theoretical grounds [75] – stasis in *itself* is not evidence for constraint [85]. After all, there is plenty of evidence of changes taking place in apparently very high-level developmental systems, even for such fundamental features as axis determination. In *Drosophila,* for example, the *bicoid*-*nanos* maternally-transcribed system is involved in the determination the initial axis laid out in the developing embryo, even though *bicoid* seems to be an innovation of the cyclorrhaphan flies to which *Drosophila* belongs [86, 87] and derived from one of the Hox genes, Hox3, which had an original role in specifying segmental identity like other Hox genes in arthropods [88-90]. At the very least, examples of conservation and change will need to be investigated in a phylogenetic perspective on a case-by-case basis before any general conclusions can be drawn. One interesting new avenue of research has been to investigate the evolution of cell types [e.g. 91], with the conclusion being that many were already present in the earliest animals. Once again though, whether this requires the early origin of complex organs too is a matter for debate.

One final point is that it is generally (although not universally!) accepted that evolution in the Cambrian was not in the style of “hopeful monsters”, with systemic changes generating whole new body plans overnight, and this is certainly not the view of [80]. Yet if not, then it turns out that the missing intermediate taxa, whether or not they have been found, must have existed at *some* point, because that would be the only way to generate new morphologies. Despite arguments that the lack of intermediates may reflect early evolutionary trajectories, I do not see any way of avoiding the conclusion that the gaps between the phyla today were generated by extinction and loss of intermediate morphologies, not because they were never there [92]. Their lack today therefore does not reflect the inability of development to generate them, but loss through competition or the great debacles of the fossil record referred to as mass extinctions.

If these (admittedly controversial) points are accepted, why, then, does the Cambrian record show the striking features it does? One possibility is from collection bias. Before the 1990s, almost all of the information of body plans present in the Cambrian came from one locality, the Burgess Shale. Since then, the extra information gained from new localities and further collections have considerably filled out the “gaps” in animal taxa. To take an example: the arthropods are now known to have evolved into several moderately diverse clades such as the anomalocaridids [93] and lamellipedians [94] by the middle of the Cambrian. Another problem is that it is generally accepted that by the time of the lower Cambrian exceptional faunas such as the Chengjiang fauna, the major clades of animals had already evolved. Unfortunately, there are simply no exceptionally well-preserved biotas known from the critical interval from about 541 to about 520 Ma [91], and it is thus very hard to assess the true patterns of diversification during this “formative interval”. Assessments of diversity and the level at which it occurs must here rely on the “conventional” small shelly fossil record, but here the taxonomy is still in need of considerable revision (see comments in e.g. [95], which remain relevant). Many clades do seem to have diversified rapidly during this time such as the molluscs [17], and although recent revisions to taxonomy in some groups such as the problematic anabaritids ([96] have greatly reduced apparent diversity, a fair number of apparently closely related taxa still seem to be present.

## SUMMARY

It is now 150 years since Salter published evidence for the “Primordial” fauna in Britain, thus effectively re-establishing the claim of the Cambrian as a geological period with its own distinctive fauna. However, he had little inkling of the vast and complex riches that the Cambrian, and now the Ediacaran, were to yield in terms of evidence for early animal evolution. What have we learnt in the meantime? The first is, as the classical exceptionally-preserved biotas have taught us, that animals had by the time of *Paradoxides* (very roughly equivalent to the age of the Burgess Shale), already diversified into most of the familiar clades of today, even though in many instances the crown group forms were yet to appear [57]. Looking further back towards the base of the Cambrian is to enter a period where the data are still relatively poor, and during this presumably critical interval we have a rather limited understanding of what sort of diversification is ongoing. Even further back during the Ediacaran, though, the data improve again, although here the problem is that they remain exceptionally challenging to interpret. It remains true, although controversial, to say that there are still no uncontested claims of crown-group cnidarians, bilaterians or even sponges from the Ediacaran, which is fairly remarkable if animals were really diversifiying just after the end of the Marinoan glaciation (c. 635 Ma). The patterns shown by Cambrian faunas have given rise to much interest and modelling concerning early developmental systems, but caution must be exercised because of the known lacunae of the critical intervals. The period of time around the Ediacaran-Cambrian boundary was a remarkable interval that changed, not just animal life, but also the planet [63], and as such can be considered to be truly revolutionary.
